# Cardiopathies congénitales: aspects épidémiologiques et échocardiographies à propos de 109 cas au centre hospitalier universitaire pédiatrique Charles de Gaulle (CHUP-CDG) de Ouagadougou, Burkina Faso

**DOI:** 10.11604/pamj.2015.20.81.5624

**Published:** 2015-01-29

**Authors:** Georges Kinda, Georges Rosario Christian Millogo, Fla Koueta, Lassina Dao, Sollimy Talbousouma, Hassane Cissé, Aristide Djiguimdé, Diarra Yé, Claudine Lougue Sorgho

**Affiliations:** 1 Unité de Formation et de Recherche en Sciences de la Santé (UFR/SDS), Université de Ouagadougou, Burkina Faso; 2 Service de Pédiatrie Médicale du CHUP-CDG de Ouagadougou, Burkina Faso; 3 Service de Cardiologie du CHU-YO de Ouagadougou, Burkina Faso; 4 Service d'Imagerie Médicale du CHU P-CDG de Ouagadougou, Burkina Faso

**Keywords:** Cardiopathie congénitale, CHUP-CDG, échocardiographie, Congenital heart disease, CHUP-CDG, echocardiography

## Abstract

Notre travail avait pour objectif d’étudier les aspects épidémiologiques et écho-cardiographiques des cardiopathies congénitales au CHUP-CDG afin d'y faire l’état des lieux. Pour se faire, nous avons mené sur une période de 27 mois d'aout 2009 à mai 2010 et d'octobre 2011 à décembre 2011, une étude rétrospective des comptes rendus d’échocardiographies Doppler des patients admis dans le service d'imagerie médicale. Nous avons utilisé une sonde cardiaque de 5MHz sur appareil Aloka Prosound 4000 Plus. Durant la période d’étude, 380 examens écho-cardiographiques ont été réalisés et ont permis de mettre en évidence 109 cas de cardiopathies congénitales avec 138 entités nosologiques différentes. Les cardiopathies congénitales représentaient 0,98% des 11169 entrées. Les souffles étaient au premier rang des motifs de demande de l’échographie Doppler cardiaque (121 cas sur 380) soit 39,53%. Les CIV étaient au premier plan des cardiopathies congénitales (28,26%), suivies des CIA (23,19%), des sténoses pulmonaires (19,57%), des Tétralogie de Fallot (9,42%). Dans leur forme isolée, les CIA étaient les plus fréquentes avec 21,95% des cas, suivies des CIV avec 20,73%. Sur 138 cas de cardiopathies congénitales (chez 109 enfants), 53 cas ont été observés chez des enfants de sexe féminin et 56 cas chez des enfants de sexe masculin soit un sexe ration de 1,1. La tranche d’âge présentant une fréquence élevée de cardiopathie congénitale est celle de 1mois- 30 mois avec 55% des cas. Les cardiopathies congénitales de l'enfant sont une réalité en Afrique, leurs fréquences dans toutes les séries rapportées sont certainement sous estimées en raison de l'inaccessibilité de l’échocardiographie doppler.

## Introduction

Les cardiopathies congénitales sont des anomalies cardiaques survenant au cours de la formation du cœur pendant la vie intra-utérine [1]. L'incidence est estimée entre 7 à 8 pour 1 000 naissances [2]. L’échocardiographie Doppler est de nos jours l'examen incontournable dans le diagnostic des cardiopathies congénitales. Au Burkina Faso, une étude rétrospective réalisée à Ouagadougou en 2006 avait rapporté une prévalence de 22,12% [3]. Elles sont donc relativement fréquentes. Une meilleure connaissance des cardiopathies congénitales de l'enfant en milieu hospitalier pédiatrique devrait permettre de proposer des mesures en vue de mieux organiser leur prise en charge; c'est l'objectif de ce travail qui vise à faire l’état des lieux des cardiopathies congénitales de l'enfant au Centre Hospitalier Universitaire Pédiatrique Charles de Gaulle.

## Méthodes

Notre travail s'est déroulé dans le laboratoire d'imagerie médicale du centre hospitalier universitaire pédiatrique Charles de Gaules de Ouagadougou pendant une période de 27 mois d'août 2009 à mai 2010 et d'octobre 2011 à décembre 2011. Il s'agit d'une étude rétrospective à visée descriptive des comptes rendus d’échocardiographies Doppler. Nous avons utilisés une sonde cardiaque de 5MHz sur appareil Aloka Prosound 4000 Plus. Tous les comptes rendus des patients âgés de 0 à 15 ans chez qui l’échocardiographie-doppler a permis de retenir le diagnostic de cardiopathie congénitale et dont le compte-rendu contenait des renseignements sur l'identité du patient, la date de réalisation de l'examen, l'indication de l'examen, les données de l'examen écho-cardiographique ont été inclus dans notre étude. La saisie et l'analyse des données ont été faites à l'aide du logiciel Epi Info version 3.4.3. L'analyse statistique a fait appel au test du Chi 2. Le seuil de significativité de p était de 0,05.

## Résultats

### Fréquence globale

**Figure 1:**
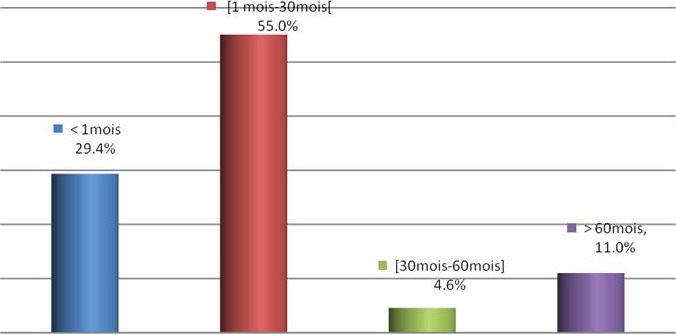
Répartition des enfants porteurs de cardiopathie congénitale en fonction de l’âge

Durant la période d’étude, 380 examens écho-cardiographiques ont été réalisés et ont permis de mettre en évidence 109 cas de cardiopathies congénitales avec 138 entités nosologiques différentes. Les cardiopathies congénitales représentaient 0,98% des 11169 entrées au CHUP-CDG. Le Tableau 1 donne la répartition des cas de cardiopathie congénitale selon les données écho-cardiographiques Certains enfants (27 cas) étaient porteurs de plusieurs types de cardiopathies congénitales associées: 11 cas de CIV + Sténose pulmonaire, 8 cas de CIV + CIA. Quatre-vingt-deux cardiopathies congénitales isolées ont été identifiées. La CIA était la cardiopathie congénitale isolée la plus fréquente avec 18 cas (21,95%), suivie de la CIV avec 17 cas soit 20,73%. Parmi les associations, la CIV associée à la sténose pulmonaire était la plus fréquente avec 40,74% suivie de la CIV associée à la CIA avec 29,63%. Le Tableau 2, nous donne la répartition nosologique des 138 cas de cardiopathie congénitale. Les CIV étaient au premier plan avec 39 cas (soit 28,26%), suivies des CIA avec 32 cas (soit 23,19%), des sténoses pulmonaires (19,57%) et de la T4F (9,42%)... L’âge moyen au moment de la réalisation de l’échocardiographie Doppler était de 5 mois avec des extrêmes de un (01) jour et 15 ans. La tranche d’âge de plus de un (01) à 30 mois était la plus représentée avec 60 cas (55%), suivie des nouveaux nés avec 32 cas (29,4%). Il n'y avait pas de différence significative dans l’âge de diagnostic des cardiopathies congénitales en fonction du sexe (X^2^= 0,33; p= 0,95). La Figure 1 nous montre la répartition des enfants porteurs de cardiopathie congénitale en fonction de l’âge. La répartition des différents types de cardiopathies en fonction de l’âge est décrite dans le Tableau 3, en éffet la majorité des cardiopathies congénitales était diagnostiquée entre 1 et 30 mois (60 cas soit 55%). En période néonatale, les CIA isolées (10 cas/18), la CIA associée à la CIV (5 cas/8), la PCA (5cas/7) ont été les cardiopathies les plus souvent diagnostiquées.

**Tableau 1:** Répartition des cas de cardiopathie congénitale selon les données écho-cardiographiques

Type de cardiopathie congénitale	Fréquence	%
Communication Inter-Auriculaire (CIA)	18	16,51
Communication Inter-Ventriculaire (CIV)	17	15,60
Sténose Pulmonaire (RP)	12	11,01
Tétralogie de Fallot (T4F)	12	11,01
CIV + RP	11	10,09
Canal Atrio-Ventriculaire (CAV)	10	9,17
CIV + CIA	8	7,34
Persistance du canal artériel (PCA)	7	6,42
Ventricule Unique (VU)	2	1,83
Hypertension Artérielle Pulmonaire (HTAP) primitive	2	1,83
CIA + RP	2	1,83
CIV + CIA + RP	2	1,83
Bicuspidie Aortique	1	0,92
T4F + Cœur Triatrial	1	0,92
CAV + CIA	1	0,92
CIA+ PCA	1	0,92
CIV + PCA	1	0,92
Maladie d'Ebstein	1	0,92
**Total**	**109**	**100,0**

**Tableau 2:** Répartition nosologique des 138 cas de cardiopathie congénitale

Entité nosologique de cardiopathie congénitale	Fréquence	%
CIV	39	28,26
CIA	32	23,19
Sténose pulmonaire	27	19,57
T4F	13	9,42
CAV	11	7,97
PCA	9	6,52
Ventricule unique	2	1,45
HTAP primitive	2	1,45
Cœur tri-atrial	1	0,72
Bicuspidie aortique	1	0,72
Maladie d'Ebstein	1	0,72
**Total**	**138**	**100**

**Tableau 3:** Répartition des différents types de cardiopathies en fonction de l’âge

	Tranche d’âge (mois)				
Type cardiopathie	< 1	[1 - 30]	]30-60]	> 60	TOTAL
CIA	10	6	1	1	18
CIV	3	11	2	1	17
Sténose pulmonaire	2	9	0	1	12
T4F	1	6	2	3	12
CIV + RP	2	9	0	0	11
CAV	1	8	0	1	10
CIV + CIA	5	1	0	2	8
PCA	5	2	0	0	7
CIA + RP	1	1	0	0	2
CIV + CIA + RP	0	2	0	0	2
HTAP primitive	0	1	0	1	2
Ventricule unique	1	1	0	0	2
Maladie d'Ebstein	0	0	0	1	1
CIV + PCA	0	1	0	0	1
Bicuspidie aortique	0	0	0	1	1
CIA + PCA	1	0	0	0	1
T4F + cœur triatrial	0	1	0	0	1
CAV + CIA	0	1	0	0	1
**TOTAL**	**32**	**60**	**5**	**12**	**109**

Nous avons noté 53 filles porteuses de cardiopathie congénitale contre 56 garçons soit un sex- ratio de 1,05. Le Tableau 4 donne la répartition nosologique des 138 cas de cardiopathies congénitales en fonction du sexe. En effet sur les 138 cas de cardiopathies congénitales diagnostiquées (observées chez 109 enfants), 66 cas ont été observés chez des enfants de sexe féminin et 72 cas chez des enfants de sexe masculin soit un sexe ration de 1,1. Le souffle cardiaque (39,53%), suivie de la détresse respiratoire (20,16%) et de la cyanose (10,9%) étaient les motifs les plus fréquents de demande de l’échographie Doppler cardiaque. Le Tableau 5 donne la distribution des motifs de demande de l’échocardiographie.

**Tableau 4:** Répartition nosologique des 138 cas de cardiopathies congénitales en fonction du sexe

Type de cardiopathie	Sexe		
	M	F	Total
CIV	23	16	39
CIA	18	14	32
RP	13	14	27
T4F	5	8	13
CAV	5	6	11
PCA	4	5	9
VU	1	1	2
HTAP	1	1	2
Bicuspidie aortique	1	0	1
Cœur triatrial	0	1	1
Ebstein	1	0	1
**TOTAL**	**72**	**66**	**138**

**Tableau 5:** Distribution des motifs de demande de l’échocardiographie

Motifs de demande	Fréquence	%
Souffle cardiaque	51	39,53
Détresse respiratoire	26	20,16
Cyanose	14	10,9
Bilan de malformation	12	9,3
Broncho-pneumopathie	12	9,3
Cardiomégalie radiologique	6	4,7
Retard de croissance	5	3,88
Infection néo natale	1	0,78
Insuffisance cardiaque	1	0,78
Précordialgie	1	0,78
**TOTAL**	**129**	**100**

## Discussion

### Limites et contraintes de notre étude

Le caractère rétrospectif de notre étude ne nous a pas permis d’être exhaustif dans la collecte des informations nécessaires à l’étude; ce qui a certainement entrainé une sous-estimation du nombre de cardiopathies congénitales. Malgré ces limites nous avons pu comparer nos résultats à d'autres auteurs et mener notre discussion

### Résultats globaux

***La fréquence***: dans notre étude, les cardiopathies congénitales occupaient 0,98% des entrées au Centre Hospitalier Universitaire Pédiatrique Charles De Gaulle. Plusieurs études rapportées par différents auteurs africains montrent des fréquences variables. Cependant, ces séries soulignent toutes de manière concordante le caractère préoccupant que revêtent les cardiopathies congénitales en Afrique. Le Tableau 6 donne les resultats comparatifs de la prévalence des cardiopathies congénitales de différentes séries africaines. Ces différences de prévalence pourraient être liées aux facteurs suivants: différence dans les critères de sélection (patients adultes) [4], séries pédiatriques dans certaines études [5–12] et enfin série échographique [6].

**Tableau 6:** Tableau comparatif de la prévalence des cardiopathies congénitales de différentes séries africaines

Etudes	Prévalence	Lieu d’étude
Kinda, Sénégal [4]	4,18%	Etudes menées en milieu cardiologique
Mayanda, Congo-Brazzaville [5]	5,09%	
Niakara, Burkina-Faso [6]	6%	
Nébié, Burkina-Faso [7]	0,72%	
Samandoulougou, Burkina-Faso [8]	0,78%	
Notre étude	0,98%	Etudes menées en milieu pédiatrique
Abena-Obama, Cameroun [9]	0,64%	
M'pemba Loufoua Lemay, Congo-Brazza [10]	0,5%	
Amon-Tanoh-Dick, Côte d'Ivoire [11]	0,1%	
Kokou Outcha, Togo [13]	0,48%	

### Répartition des patients selon le sexe

Le sexe masculin prédominait dans notre étude avec un sex-ratio à 1,1. Ceci rejoint les résultats de Kokou, Kinda, Ould, Touré, Diop, M'pemba-Loufoua, Acrachi [4, 10, 12–16] qui notaient une prépondérance du sexe masculin avec respectivement un sex-ratio de 1,6; 1,5; 1,3; 1,16; 1,11; 1,09; 1,07. Pour la plupart des auteurs, le sexe n’était pas incriminé dans la genèse des cardiopathies congénitales et il n'existait pas de prédominance nette.

### Répartition des patients selon l’âge

L’âge moyen des enfants dans notre série était de 5 mois avec des extrêmes de 1 jour et 15 ans. La tranche d’âge de 1 à 30 mois était la plus représentée avec 60 cas (55%); 84,24% des cardiopathies congénitales ont été diagnostiqués entre 0 et 30 mois. Ceci est en accord avec Abéna [9] et Cloarec [17] qui ont remarqué que la plupart des cardiopathies congénitales était diagnostiquée dès le bas âge entre 0 et 2 ans respectivement 70% et 61%. Ce taux élevé des cas de cardiopathie congénitale dans la tranche d’âge de 1à 30 mois par rapport aux âges de plus de 30 mois dans notre étude s'expliquerait par le fait que: Certaines cardiopathies congénitales comme les CIV, les CIA et la PCA qui représentaient 57,97% des cas dans notre étude ont une évolution spontanée vers la fermeture [18–20]; les cardiopathies congénitales de découverte tardive sont le plus souvent bénignes et compatibles avec une vie quasi normale. Alors ces patients ne consultent pas et ne sont souvent pas recensés.

### Les indications de l’échocardiographie

Les souffles étaient au premier rang des motifs de demande de l’échographie Doppler présent dans 39,53% des cas dans notre série, suivie de la détresse respiratoire 20,16%. Ces résultats pourraient s'expliquer par le fait que le souffle est un signe quasi constant dans les cardiopathies congénitales chez les enfants [21]. En ce qui concerne la détresse respiratoire, les infections respiratoires récurrentes sont de fréquentes circonstances révélatrices des cardiopathies congénitales [9, 12] favorisées par des facteurs locaux (mauvaise hygiène, étroitesse des voies aériennes dans les syndromes poly-malformatifs) et généraux (inondation et hypersécrétion pulmonaire, déficit immunitaire complexe).

### Les différentes cardiopathies rencontrées

La cardiopathie la plus fréquemment retrouvée dans notre étude et dans la plupart des études en Afrique [4, 9, 11, 13–16, 22–25] et dans le monde [19, 26] était la CIV. Le Tableau 7 donne les résultats comparatifs de la fréquence de la CIV en Afrique et dans le monde. Elle est unique (avec prépondérance de la forme péri-membraneuse: 98,4%) ou associée à d'autre cardiopathies; dans ce cas, c'est l'association avec la sténose pulmonaire qui était la plus fréquente avec 11 cas. Ces résultats sont similaires à ceux de Sanogo et Sawadogo [3, 27] au Burkina-Faso et à ceux de Glen et al. [28] en Angleterre. La prédominance de cette association pourrait s'expliquer par le fait que la sténose pulmonaire représente une évolution anatomique des CIV comme il est décrit dans la littérature [21].

**Tableau 7:** Tableau comparatif de la fréquence des CIV dans le monde

Pays	Auteurs	Fréquence CIV (%)
Togo	Kokou Outcha [13]	24,4
Sénégal	Acrachi [12]	38
Sénégal	Kinda [4]	23,33
Burkina-Faso	Sanogo [3]	37
Burkina-Faso	Sawadogo [27]	50
France	Joly et al. [19]	30 à 40
Etats-Unis	Sables [26]	30
Burkina-Faso	Notre étude	28,26

## Conclusion

Ce travail rétrospectif a permis de confirmer que les cardiopathies congénitales de l'enfant sont une réalité avec une fréquence de 0,98% des entrées au Centre Hospitalier Universitaire Pédiatrique Charles De Gaulle de Ouagadougou. Cette fréquence est probablement sous-estimée en raison de l'irrégularité de la disponibilité de l’échocardiographie au CHUP-CDG. Notre étude ayant considéré uniquement les aspects épidémiologiques et écho-cardiographiques des cardiopathies congénitales chez l'enfant au CHUP-CDG, d'autres études plus représentatives méritent d’être réalisées dans le but de déterminer leur prévalence au Burkina-Faso, pour une meilleure organisation prise en charge de cette pathologie au CHUP-CDG de Ouagadougou.
